# Exploring perinatal mental well-being: a concept analysis from conception to one year postpartum

**DOI:** 10.1186/s12884-025-08015-y

**Published:** 2025-10-10

**Authors:** Mia Massaer, Erik Franck, Sarah Van Haeken, Annick Bogaerts, Olaf Timmermans

**Affiliations:** 1 Faculty of Medicine and Health Sciences, Centre for Research and Innovation in Care (CRIC), University of Antwerp, Campus Drie Eiken, Gebouw S, Universiteitsplein 1, WilrijkAntwerp, 2610 Belgium; 2 Research & Expertise, Resilient People, UC Leuven-Limburg, Quadrigebouw, Wetenschapspark 21, Diepenbeek, 3590 Belgium; 3 Faculty of Medicine, Department of Development and Regeneration, Women and Child, KU Leuven, Building ‘Onderwijs en Navorsing 2’, Herestraat 49, Louvain, 3000 Belgium; 4 Research Group Healthy Region, HZ University of Applied Sciences, Edisonweg 4, Vlissingen, 4382 NW Netherlands

**Keywords:** Concept analysis, Perinatal mental well-being, Prenatal mental health, Postnatal mental health, Perinatal psychological well-being, Perinatal emotional well-being, Perinatal social well-being, Positive mental health, Maternal mental health, Midwifery

## Abstract

**Background:**

Perinatal Mental Well-Being (PMWB) is critical for the health of both mothers and children. Despite its importance, a clear, objective, and measurable definition remains lacking. This study aimed to explore and define PMWB from conception to one year postpartum.

**Methods:**

A concept analysis was conducted using Walker and Avant's framework. A comprehensive literature search was performed across various sources, including dictionaries, thesauri, encyclopaedias, and textbooks related to General Mental Well-Being, midwifery, psychology, and mental health journals, alongside international databases. The search was performed without date restrictions and included publications up to November 2024. The primary criterion for inclusion was relevance to the definition of PMWB. Data were analysed using the constant comparison method and managed with MAXQDA Analytics Pro (24.4.1). Identified meaning units were coded into codes and subcodes based on the attributes of PMWB. The review included twenty articles, yielding 241 extracted codes, which were subsequently categorized into key attributes and subdimensions of PMWB. The methodological quality of the included studies was assessed using Joanna Briggs Institute (JBI) tools or alternative criteria, depending on study type. The analysis showed that PMWB has three key aspects: Emotional (Hedonic), Psychological (Eudaimonic), and Social Well-Being, each with unique characteristics. PMWB is also intricately connected to other dimensions of the General concept of Perinatal Well-Being, including Physical, Spiritual, Economic, and Ecological factors. These connections highlight the complexity and dynamic nature of PMWB.

**Conclusion:**

PMWB is a multidimensional concept that integrates emotional, psychological, and social domains and extends to other dimensions such as Physical, Spiritual, and Economic Well-Being. This holistic perspective captures the diverse scope of maternal mental health in the perinatal period. Given the evolving nature of PMWB, detailed and adaptable assessment tools are crucial for reflecting women's experiences and guiding timely interventions to improve long-term outcomes for mothers and their children.

**Supplementary Information:**

The online version contains supplementary material available at 10.1186/s12884-025-08015-y.

## Background

Perinatal Mental Well-Being (PMWB) is increasingly recognised as a cornerstone of maternal and child health, from conception to the first year postpartum [1–7]. During this critical period, the mother's mental health not only influences her own Well-Being (WB) but also profoundly impacts her child’s physical, cognitive, and emotional development [2, 8]. Maternal mental health disorders, including depression and anxiety, have been linked to insecure mother–child attachment, higher risks of child neglect, and a greater prevalence of physical and behavioural issues in children [6, 7, 9–11]. These findings underscore the necessity of integrated support systems to safeguard and enhance women’s mental health throughout the perinatal period [12, 13].

Although often associated with pregnancy and postpartum, the perinatal period begins at conception. This early stage can already trigger strong emotional responses, identity shifts, and psychological adjustments. From the moment a woman becomes aware of her pregnancy, she may experience joy, anxiety, or ambivalence. These early reactions are part of the evolving Mental Well-Being of expectant mothers. Including conception in the scope of Perinatal Mental Well-Being allows for a more comprehensive understanding of maternal psychological needs [14, 15].

### Impact of PMWB on mother health and barriers

Given the considerable influence of PMWB on maternal and child health outcomes, there is an urgent need for governmental and policy-level support, interventions to optimise PMWB, and preventive measures against mental health issues. Around 1 in 5 women experience mental health issues, such as anxiety and depression, during the perinatal period [1, 2, 16–19]. Most of the present knowledge about PMWB comes from studies on negative affect and mental disease. It is time to rethink this concept, shifting the focus away from negative mental outcomes and towards optimising and enhancing PMWB. This concept should not be seen as merely the absence of psychological distress. Instead, it refers to a dynamic and multidimensional state of emotional, psychological, and social flourishing. This broader perspective is supported by Keyes, who defines mental health as a complete state that combines the absence of illness with the presence of Well-Being [20]. His model invites a shift towards a strengths-based view of PMWB, where positive functioning becomes central. Barry expands on this by highlighting how mental health can be actively promoted. She points to resilience, social connectedness, and self-management as key elements, particularly within maternal care and public health policy [21, 22]. Ryff adds another layer with her eudaimonic perspective, which emphasises meaning, autonomy, and personal growth as essential to psychological Well-Being—elements that are especially relevant during the perinatal period [23].

The World Health Organization has also advocated for a more health-promoting approach to mental health. Rather than focusing solely on the treatment of illness, WHO promotes supportive environments that foster Well-Being, particularly in maternal and child healthcare [3–5]. These perspectives not only promote a strengths-based understanding of PMWB but also align with principles of preventive care, emphasizing early support and sustained Mental Well-Being throughout the perinatal period.

Without proactive approaches, mental health issues in the perinatal period often remain undiagnosed, reinforcing the saying"there is no health without mental health" [2, 21, 24]. Despite its importance, up to 50% of perinatal depression cases remain unrecognised and untreated in midwifery and obstetric settings [25, 26]. This makes perinatal depression the most underdiagnosed and untreated obstetric complication [25–27]. This oversight results in significant suffering for women and their families, making their decline in health and PMWB greater than it should be. At the same time, the economic costs are high [28–31]. Due to loss of productivity, increased expenditure on maternal health and obstetric-specific expenditures. In addition to the tax burden, maternal suicide is a leading cause of maternal mortality, with suicide rates peaking in the postpartum period [32, 33]. The stigma associated with mental health remains fundamental in the barriers to seeking help​ [25, 28, 30, 31, 34, 35].

### Role of midwifery in supporting PMWB

Enhancing maternal mental health has the potential to support early childhood developmental outcomes and reduce long-term healthcare costs associated with mental health treatment. The perinatal period plays a critical role in the health of both mother and child. Including this concept in public health strategies may promote healthier families and improved child development. This approach benefits overall public health [1]. Routine antenatal care needs to include optimisation of PMWB and psychosocial support. There is a gap in early detection, screening, and support [36]. Midwives, the lead professionals in perinatal care, have frequent contact with women and can educate, support, and identify at-risk people. Midwifery care, based on principles of continuity of care, woman-centred care, informed choice, and advocacy, may facilitate uptake in perinatal mental health care [28, 37]. Currently, there is no consensus on the core components of PMWB, and there is also no established'gold standard'for its measurement.

Despite increasing attention to PMWB in research and practice, its conceptualisation remains ambiguous and inconsistently applied [14, 15]. Terms such as “maternal mental health,” “Well-Being,” and “perinatal emotional health” are often used interchangeably, leading to confusion about what PMWB entails [14]. Moreover, existing measurement tools tend to focus on the absence of distress rather than on the presence of positive mental states [21]. This lack of conceptual clarity limits the development of effective screening instruments, hinders communication between professionals, and prevents the design of targeted interventions. Therefore, a concept analysis is necessary to define the core attributes, antecedents, and consequences of PMWB [38, 39]. It provides a theoretical foundation to better understand, assess, and promote maternal mental health during the perinatal period.

### Objectives

This study aims to address challenges in defining and measuring PMWB by exploring its dimensions and proposing tools for tailored support.

## Methods

This review utilised the concept analysis approach outlined by Walker and Avant [38]. This is a method of concept analysis that borrows from linguistic philosophy ways of reducing complex concepts into simpler forms and breaking them down. It is particularly suited for exploring complex constructs such as PMWB, where poorly defined use of terms can result in apparently inconsistent communication among researchers and clinicians [38]. The method identifies the core attributes, antecedents, and consequences involved in the definition refinement process and gives helpful guidance regarding its application within maternal health contexts [39].

This structured approach has the advantage of clarity about PMWB, which is particularly necessary when constructs within the field are not standardised. Identifying empirical referents is essential, establishing measures of PMWB as reliable and valid. This is a critical step in developing evidence-based interventions within perinatal care. conceptualisation is best viewed, however, as an evolving process; meaning knowledge that is changing rather than a static and immutable entity [40].

## Search strategy

An extensive literature search was conducted across various databases and search engines without restrictions on the publication date, up until November 2024. The databases included PubMed, Cochrane, Trip Medical Database, PsycInfo, Google Scholar, CINAHL, Medline via OVID, and Web of Science. Grey literature was included in the search strategy through Google Scholar and manual snowballing using reference lists. However, only sources that met the predefined inclusion and quality criteria were retained for the final analysis. The search strategy involved a combination of keywords and MeSH (Medical Subject Headings) terms, combined with individual keyword searches, to ensure comprehensive coverage.

The complete search strategy is summarised in Table 1.

**Table 1:** Search strategy and search terms used in the literature review

Search Component	Details
Databases searched	PubMed, Cochrane, Trip Medical Database, PsycInfo, Google Scholar, CINAHL, Medline via OVID, Web of Science
Search period	No restriction on publication date; search performed until November 2024
Search method	Combination of keywords and MeSH (Medical Subject Headings) terms, both individually and in combination
MeSH terms and keywords (combined with AND)	- Perinatal Care [MeSH] OR Prenatal Care [MeSH] OR Postnatal Care [MeSH] OR Peripartum Period [MeSH] OR Antenatal Care [MeSH] OR Pregnancy [MeSH] - AND Mental Health [MeSH] OR Mental Well-Being OR Psychological Well-Being [MeSH] OR Emotional Well-Being OR Social Well-Being [MeSH] OR Maternal Well-Being [MeSH] OR Hedonic Well-Being OR Eudaimonic Well-Being
Additional terms	Well-Being [MeSH], Quality of Life [MeSH]
Exclusion terms	NOT (Paternal [MeSH] OR Foetal Loss [MeSH] OR Contraception [MeSH] OR Abortion, Induced [MeSH])
Additional methods	Manual searching of reference lists, dictionaries, thesauri, encyclopaedias, midwifery and psychology journals, mental health literature, and Well-Being textbooks

To ensure conceptual completeness, additional keyword searches were conducted individually to capture a broad range of studies and definitions related to “Mental Well-Being,” “Well-Being,” and similar concepts. The reference lists of retrieved articles were manually screened for further relevant literature. Grey literature was also consulted, including dictionaries, thesauri, encyclopaedias, midwifery and psychology journals, and Well-Being textbooks. These sources helped define PMWB, identify relevant synonyms, and integrate theoretical perspectives and conceptual frameworks from related disciplines.

## Eligibility criteria

Eligibility criteria are summarised in Table 1. Studies were included if they provided a conceptual or theoretical perspective on Perinatal Mental Well-Being (PMWB), Mental Well-Being (MWB), or General Well-Being (GWB), with particular attention to the mental dimension of well-being. Studies were excluded if they focused exclusively on paternal Well-Being, contraception, abortion, or foetal loss. Only peer-reviewed publications in English were considered.

## Data extraction and analysis

Data extraction was performed by a single author using a structured form. This form was designed to capture key study elements, such as the study aim, definitions related to PMWB, and participant criteria. The extracted data were then synthesised narratively to construct a detailed profile of PMWB definitions, as reported in the included studies.

Data analysis followed the eight-step concept analysis approach described by Walker and Avant to ensure a systematic exploration of the concept of PMWB [38]. This method provided a comprehension of PMWB through the following steps: selecting the concept, defining the aims of the analysis, exploring its applications, identifying its defining attributes, developing a model case, distinguishing borderline and related cases, determining the concept's antecedents and consequences and finally establishing empirical referents.

Qualitative data analysis using MAXQDA Analytics Pro (version 24.4.1), enhanced this process allowing for coding and categorising data into dimensions and subdimensions.

The analysis utilised the four-step Constant Comparison method. First, relevant excerpts were coded and systematically organised using MAXQDA Analytics Pro software. Second, the data were visualised in tables to facilitate straightforward analysis. Third, similar codes were grouped into subcategories, which were subsequently aggregated into broader categories. Finally, conclusions were drawn to ensure a clear understanding of the attributes of PMWB, as summarised in Tables 2 and 3.

### Quality assessment

The methodological quality of the included studies was assessed using the appropriate Joanna Briggs Institute (JBI) Critical Appraisal Tools, tailored to each study design. Where standard tools were not applicable, particularly for conceptual and theoretical contributions, alternative criteria such as clarity of argumentation, theoretical consistency, and relevance to the conceptual aim of the analysis were used. Overall, the included studies demonstrated moderate to high methodological quality. Empirical studies generally met key criteria related to recruitment, measurement, and analysis, while systematic reviews showed sufficient transparency in their search and selection strategies. Conceptual articles were judged to offer substantial theoretical insight and relevance, despite the absence of standardised appraisal frameworks. No articles were excluded based on quality, as the primary criterion for inclusion was their conceptual relevance to the aim of the analysis. A complete overview of the quality appraisal of all included articles is provided in Additional File 2: Quality Appraisal Summary.

## Results

### Search and screening process

**Fig. 1:**
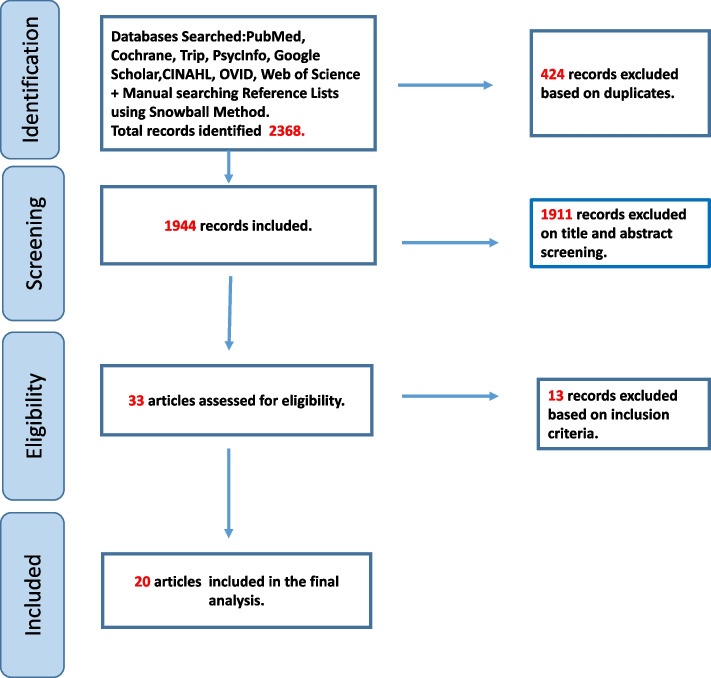
Screening process flow chart

An advanced literature search was conducted, retrieving a total of 2,368 records from the original search strategy, including results from databases such as PubMed, Cochrane, Trip, PsycInfo, Google Scholar, CINAHL, OVID, and Web of Science, as well as through manual searching using reference lists and the snowball method. After removing 424 duplicates, 1,944 records were included for title and abstract screening. Following this screening process, 1,911 records were excluded for not meeting the relevance criteria. Subsequently, 33 full-text articles were assessed for eligibility. Specifically, articles were excluded if they lacked a clear conceptual or theoretical focus on Mental Well-Being or did not provide sufficient detail to support concept analysis. Of these, 13 articles were excluded for failing to meet the inclusion criteria. Ultimately, 20 articles [14, 15, 41–48], [3–5, 20, 21, 23, 49–55] were included in the final analysis. This systematic approach ensured a comprehensive and unbiased review of the topic, as illustrated in Fig. 1.

A detailed overview of the included studies is available in Additional file 1 (see Additional file 1). Among the 20 articles retained for this analysis, four reports from the World Health Organization (WHO, 1946, 2004, 2012, 2018) [3–5, 51] were included as a single entry in Additional file 1 of the included articles. This decision was made to avoid redundancy in reporting, as the WHO reports share consistent themes and a unified emphasis on multidimensional Well-Being. However, during the qualitative coding process, these four reports were treated as separate sources to ensure that their unique contributions and nuanced perspectives were adequately captured in the analysis.

## Methodological steps in concept analysis

### Step 1: selection of a concept

For this analysis, Perinatal Mental Well-Being (PMWB) was chosen as the focal concept because it plays a pivotal role in the health and Well-Being of both mothers and infants along the continuum of the perinatal period [24]. PMWB addresses maternal mental health challenges during sensitive transitions when both maternal and infant health outcomes are particularly vulnerable [15]. Despite its critical importance, PMWB remains under-researched and lacks a universally accepted definition. The term is often used interchangeably with broader concepts like Mental Well-Being (general concept) or Maternal Health, making it challenging to distinguish its unique dimensions [14, 56]. This ambiguity can hinder healthcare providers’ ability to identify at-risk mothers and design targeted interventions [28, 43, 57].

Clarifying PMWB as a concept plays a crucial role in the advancement of maternal mental health practices [49]. It also supports the development of evidence-based tools and care plans tailored for healthcare professionals, especially midwives and nurses [10, 26, 51]. Moreover, a well-defined conceptual framework helps to foster more holistic approaches to maternal health [27, 30].

### Step 2: determine the aims or purposes of the analysis

The primary purpose of this concept analysis is to establish an operational definition of PMWB, providing a foundation for developing reliable measurement tools. Current evidence demonstrates that maternal mental health profoundly impacts prenatal and postnatal outcomes, including infants’ physical, emotional, and cognitive development [6, 7].

This analysis seeks to achieve three main objectives. First, it aims to define the core attributes, antecedents, and consequences of PMWB in order to guide effective interventions [38]. Second, it strives to create a robust framework that healthcare providers, particularly midwives, can apply to enhance maternal care practices [58, 59]. Third, it intends to bridge the gap between research and clinical application by offering practical tools to identify women's needs across the PMWB spectrum [43, 57].

By addressing these aims, midwives and other professionals will be better equipped to identify and support women at risk of poor mental health during the perinatal period, ultimately contributing to improved long-term maternal and child outcomes [58, 59].

### Search overview

An advanced literature search across multiple databases and grey literature sources yielded a total of 2,368 records. After removal of duplicates and a two-step screening process (title/abstract and full text), 20 articles were retained for final concept analysis. A detailed overview of this process is presented in the Results section and illustrated in Fig. 1.

### Step 3: Identify all uses of the concept

WB is a multidimensional concept that is universally accepted and applied in various domains [60–62]. MWB is a core dimension of the concept GWB. Although not widely used in other areas, PMWB plays a significant role in addressing the mental health issues women experience during the perinatal period [15].

Conceptualisation of women's Perinatal Well-Being indicates that it is simply abstract and multifaceted, entailing self-assessment related to different dimensions of life within the perinatal period itself [15]. Wadephul et al. (2020) conducted a systematic review according to the overarching construct of Perinatal Well-Being (PWB) and reported three key themes [15]. PWB was defined as a subjective experience encompassing physical, affective, and psychological/cognitive dimensions. This suggests that PWB is an entirely individualistic concept because everyone is unique compared to all others. Finally, they viewed PWB as dynamic, evolving due to the influence of modifiable factors during the perinatal period [15].

In midwifery, PMWB is increasingly viewed not only as identifying and managing mental health problems but also as promoting positive mental health outcomes. Traditionally, PMWB focused on preventing and treating conditions like depression and anxiety. However, there is growing emphasis on enhancing PMWB as part of holistic maternity care [63–65]. Midwives play a critical role in this broader strategy by prioritising both maternal and child Well-Being, encompassing physical, emotional psychological, and social dimensions. Continuity of midwifery care fosters trusting relationships, enabling women to share mental health concerns openly and build resilience during the perinatal period [63–65].

Broader definitions of WB, such as the World Health Organization’s, emphasise mental, physical, and social dimensions as centric to overall health [66]. Within this model, PMWB is closely aligned with this consensus and illuminates the need for a comprehensive approach throughout the perinatal period [1, 6]. While PMWB shares conceptual space with the idea of MWB (the general concept), it uniquely includes factors specific to the perinatal period. Included among these factors are changes in hormones, physical health problems linked to the state of being pregnant, and the psychological anticipation of parenthood, which together strengthen maternal mental resilience [6, 15]. It is important to note that PMWB is not static but rather dynamic, changing throughout the perinatal period [66]. Factors like hormonal shifts and varying levels of social support can significantly influence a woman’s mental state during this time.

Additionally, cultural and socio-economic contexts shape how women experience PMWB. For instance, societal expectations and living conditions influence stress levels and access to resources. Addressing these diverse needs requires a context-sensitive approach to PMWB [65].

### Step 4: defining attributes of the concept PMWB

#### Core dimensions of PMWB

PMWB is a dynamic, multifaceted concept that includes a subjective positive self-evaluation across three primary subdimensions:

- Emotional WB: This dimension includes the ability to experience positive feelings, maintain mood stability, and find life satisfaction and happiness throughout the perinatal period.
- Psychological WB: Psychological WB during the perinatal period encompasses a sense of personal growth, autonomy, purpose in life, self-acceptance, all of which are essential as women transition into motherhood.
- Social WB: Social WB reflects the mother’s sense of social coherence, integration, and connectedness within her community, as well as a feeling of meaningful contribution and belonging in her social environment during the perinatal period.

#### Interconnection with other well-being dimensions

PMWB is intricately connected to other dimensions of the broader WB concept, including:

- Physical WB: The physical health of the mother, including factors such as nutrition, exercise, and the absence of chronic illness.
- Spiritual WB: A sense of meaning, purpose, and existential fulfilment, which can be particularly significant during pregnancy and the transition to motherhood.
- Economic WB: Financial stability and access to resources that reduce stress and enable better health outcomes.
- Ecological WB: The impact of the environment, including access to clean air, water, and safe living conditions.

These dimensions are interrelated and mutually influence one another, contributing to a holistic state of WB during the perinatal period.

#### Temporal Scope of PMWB

The perinatal period is from conception through 12 months postpartum. This period is marked by significant physical, emotional, psychological, and social changes, crucial for defining and assessing PMWB.

The core dimensions and key elements of PMWB identified in this analysis are presented in Table 2. The analysis provides a detailed breakdown of the qualitative coding process, summarised in Table 3, which quantifies the frequency of explicit (EX), implicit (IM), and not mentioned (NM) codes for the identified attributes. This table builds on the framework presented in Table 2 by offering a quantitative perspective on the core dimensions and their connections to broader Well-Being domains. For instance, Emotional attributes such as ‘Positive Feelings’ and ‘Mood Stability’ are prominently represented, with frequent explicit mentions in the analysed literature. Conversely, dimensions like ‘Social Coherence’ and ‘Ecological Well-Being’, are less emphasised, as evidenced by their relatively low frequency of explicit mentions. Spiritual Well-Being, which captures meaning and existential fulfilment, was explicitly mentioned in only five studies. Economic Well-Being, encompassing financial stability and access to resources, appeared more frequently, with six explicit and ten implicit mentions.

Table 2 presents the conceptual framework of Perinatal Mental Well-Being (PMWB), outlining its core dimensions, antecedents, and consequences. This framework illustrates the interconnections between Emotional, Psychological, and Social Well-Being, while integrating other dimensions of Well-Being. Additionally, the table highlights key attributes and empirical referents, identifying both prominent aspects and gaps in representation, underscoring the need for a holistic approach to PMWB in future research (see Table 2).

Following the conceptual framework outlined in Table 2, a detailed breakdown of the qualitative coding process is presented in Table 3. This table quantifies the frequency of explicit (EX), implicit (IM), and not mentioned (NM) codes for the identified attributes, providing a data-driven perspective on the key dimensions of PMWB (see Table 3).

**Table 2:** Conceptual framework for perinatal mental well-being (PMWB)

Category	Core Dimensions	Key Elements	Description
**Defining Attributes of PMWB**	**Emotional Well-Being**	• Positive feelings • Mood stability • Life satisfaction • Happiness	Ability to experience positive emotions, maintain mood stability, and find satisfaction and happiness
	**Psychological Well-Being**	• Personal growth • Autonomy, • Purpose in life • Self-acceptance	Encompasses a sense of personal growth, autonomy, purpose in life, self-acceptance, all essential as women transition into motherhood
	**Social Well-Being**	• Connectedness • Integration • Social coherence	Reflects the mother’s sense of social coherence, integration, and connectedness within her community, as well as feeling a meaningful contribution and belonging
**Integration with other Well-Being Dimensions**		**Key Elements**	
**Antecedents of PMWB**	Physical Well-Being	Nutrition, exercise, absence of chronic illness	
	Spiritual Well-Being	Purpose, meaning, existential fulfillment	
	Economic Well-Being	Financial security, access to resources	
	Ecological Well-Being	Safe environment, access to clean air and water	
	**Core Dimensions**	**Key Elements**	**Description**
	Social Support	Support from family, friends, caregivers	Social support reduces stress and promotes mental Well-Being during the perinatal period
	Economic Stability	Financial security, access to essential resources	Financial stability reduces stress and ensures access to essential resources
	Access to Healthcare	Regular prenatal and postnatal care	Healthcare enables early detection and management of mental health concerns
	Education and Awareness	Knowledge of mental health needs, prenatal classes, early intervention	Education builds skills to manage mental health and enhance resilience
	Physical Health	Good physical health, absence of chronic conditions	Physical health supports energy, resilience, and Well-Being during the perinatal period
	Psychosocial Resilience	Coping strategies, resilience, positive outlook	Resilience and coping strategies aid in managing perinatal challenges
	Cultural and Environmental Factors	Supportive cultural practices, access to green spaces, safe living environment	Supportive culture and environment enhance Perinatal Mental Well-Being
**Consequences of PMWB**	**Core Dimensions**	**Key Elements**	**Description**
	Mother-Infant Bonding and Child Development	Psychological attachment between mother and child	Strengthens the mother-infant bond, fostering the child’s cognitive, emotional, and social development
	Impact on Maternal and Infant Health	Reduced mental health risks and better outcomes	Improves maternal mental health and reduces the risk of postnatal depression, leading to healthier outcomes for both mother and child
	Healthcare Cost Reduction	Efficient resource use	Reduces healthcare costs by minimizing the incidence of mental health issues
	Stable Family Relationships	Strengthened relationships between partners	Fosters a supportive family environment, reducing relational stress and improving child development
	Long-Term Resilience	Sustained health and resilience	Builds long-term adaptability and resilience in both mother and child, with benefits for broader public health
**Empirical Referents**	**Core Dimensions**	**Key Elements**	**Description**
	Capture My Mood (CMM) Scale	Real-time tracking of emotional and psychological states	A self-monitoring tool that correlates with the WEMWBS, useful for tracking Well-Being during the perinatal period
	Well-Being in Pregnancy (WiP) Questionnaire	Assessment of mental Well-Being during pregnancy	Designed for monitoring mental Well-Being, particularly in women with long-term health conditions
	Warwick-Edinburgh Mental Well-Being Scale (WEMWBS)	Benchmark Well-Being scale	A validated scale for measuring overall mental Well-Being, commonly used as a benchmark

This table presents the perinatal mental Well-Being (PMWB) conceptual framework. It outlines the core dimensions, key elements, and descriptions of PMWB. The interconnection with the other dimensions of the general concept of Perinatal Well-Being is also explained

The table also highlights key antecedents, consequences, and empirical references, providing a structured approach to understanding PMWB and its impact on maternal and child health

The attributes presented in this table were identified through a systematic concept analysis using MAXQDA Analytics Pro, synthesizing 241 codes from 20 articles into key attributes and subdimensions such as Emotional, Psychological, and Social Well-Being, as well as their interconnections with broader Perinatal Mental Well-Being (PMWB) factors

Of the 20 articles retained for this concept analysis, four key documents from the WHO (1946, 2004, 2012, 2018) were coded as separate sources during the qualitative analysis. This approach ensured that the unique contributions of each document were accounted for. However, due to their shared themes and consistent emphasis on multidimensional Well-Being, these documents were later grouped as one entry in the final summary table to avoid redundancy in reporting

**Table 3:** Summary of attributes and references for perinatal mental well-being coding

Categories	Subcodes	Total EX Explicit Mentions	Total IM Implicit Mentions	Total EX + IM	Total NM Not Mentioned	References EX Explicit Mentions	References IM Implicit Mentions	References NM Not Mentioned
**Emotional Well-Being**	Mood stability	7	14	21	2	[14, 15, 41–45]	[3, 4, 20, 21, 23, 46–54]	[5, 55]
	Positive feeling	21	2	23	0	[3–5, 14, 15, 20, 21, 23, 42–50, 52–55]	[3–5, 14, 15, 20, 21, 23, 42–50, 52–55]	
	Life satisfaction	17	6	23	0	[3, 4, 15, 20, 21, 23, 42, 43, 46–50, 52–55]	[14, 18, 41, 44, 45, 51]	
	Happiness	17	6	23	0	[4, 15, 20, 21, 23, 42–50, 53–55]	[5, 14, 16, 41, 51, 52]	
**Psychological Well-Being**	Personal Growth	8	15	23	0	[3–5, 15, 20, 23, 49, 55]	[14, 21, 41–48, 50–54]	
	Autonomy	16	5	21	2	[4, 15, 20, 21, 23, 41, 44–49, 52–55]	[3, 5, 42, 43, 50]	[14, 51]
	Purpose in life	16	6	22	1	[4, 5, 14, 15, 21, 23, 42–45, 48, 49, 52, 54, 55]	[2, 3, 46, 47, 50, 53]	[51]
	Self-acceptance	7	14	21	2	[15, 20, 23, 47–49, 55]	[3–5, 14, 21, 41–45, 50, 52–54]	[46], [51]
**Social Well-Being**	Connectedness	19	4	23	0	[3–5, 14, 15, 20, 21, 23, 41, 44–48, 50, 52–55]	[42, 43, 49, 51]	
	Integration	5	18	23	0	[3–5, 15, 46]	[14, 20, 21, 23, 41–45, 47–55]	
	Social coherence	2	9	11	12	[4, 55]	[3, 5, 21, 23, 41, 46, 50, 52, 53]	[14, 15, 20, 42–45, 47–49, 51, 54]
**Integration with Other Dimensions**	Physical Well-Being	19	3	22	1	[4, 5, 15, 20, 21, 23, 43–48, 51–55]	[41, 42, 50],	[49]
	Spiritual Well-Being	5	11	16	7	[15, 20, 43, 46, 48]	[3–5, 14, 21, 23, 47, 49, 50, 52, 54]	[41, 42, 44, 45, 51, 53, 55]
	Economic Well-Being	6	10	16	7	[3–5, 21, 47, 52]	[14, 15, 20, 23, 44, 45, 48, 50, 53, 54]	[41–43, 46, 49, 51, 55]
	Ecological Well-Being	0	6	6	17		[4, 5, 15, 21, 43, 47]	[3, 14, 20, 23, 41, 42, 44–46, 48–55]
**Multifaceted**	Multifaceted	19	2	21	2	[3–5, 14, 15, 20, 21, 23, 41, 42, 44–48, 50, 52–54]	[3, 49]	[51, 55]
**Dynamic**	Dynamic	16	6	22	1	[4, 5, 14, 15, 21, 23, 41, 43–46, 48, 52–55]	[3, 20, 42, 47, 49, 50]	[51]
**Subjective positive self-evaluation**	Positive Self-Reflection	9	13	22	1	[4, 5, 14, 15, 20, 41, 43, 48, 52]	[3, 21, 23, 42, 44–47, 49, 50, 53–55]	[51]

EX refers to explicit attributes that were directly mentioned in the analysed texts. IM represents implicit attributes, which were inferred from contextual cues within the texts. The notation (EX + IM) indicates the combined count of both explicit and implicit mentions. NM refers to attributes that were not mentioned in the analysed texts

The attributes presented in this table were identified through a systematic concept analysis using MAXQDA Analytics Pro, synthesizing 241 codes from 20 articles into key attributes and subdimensions such as Emotional, Psychological, and Social Well-Being, as well as their interconnections with broader Perinatal Mental Well-Being (PMWB) factors

Of the 20 articles retained for this concept analysis, four key documents from the WHO (1946, 2004, 2012, 2018) were coded as separate sources during the qualitative analysis. This approach ensured that the unique contributions of each document were accounted for. However, due to their shared themes and consistent emphasis on multidimensional Well-Being, these documents were later grouped as one entry in the final summary table to avoid redundancy in reporting

### Step 5: model case

The model case presented in Table 4 was developed based on key findings from the concept analysis.

**Table 4:** Cases analysis of perinatal mental well-being

	**Model Case of PMWB**
**Context**	Jane is a 30-year-old woman in her third trimester of pregnancy. She is usually in good health, and this is her first pregnancy. Jane is living in a caring place with a partner who is involved in the pregnancy. Her family and friends are very close to her and help her in both mental and practical ways
**Antecedents**	**Social Support:** Jane’s partner attends prenatal appointments with her. They both participate in a local parent support group where they discuss their experiences with other expectant parents **Economic Stability:** Jane and her partner have stable jobs, which allows them to comfortably afford necessary prenatal care, nutrition, and preparation for the baby’s arrival **Access to Healthcare:** Jane has regular check-ups with her midwife, who provides comprehensive prenatal care and discusses both Physical and Mental Well-Being with her **Education and Awareness:** Jane has participated in prenatal classes where she learned about the and emotional changes during pregnancy, as well as strategies for maintaining Mental Well-Being. **Physical Health:** Jane is in good physical health, with no chronic conditions. She maintains a balanced diet and engages in regular exercise to support her pregnancy **Psychosocial Resilience**: Jane demonstrates strong psychological resilience by using effective coping strategies and maintaining a positive outlook. She feels confident in her ability to navigate the changes and challenges of the perinatal period **Cultural Factors:** Jane feels strengthened by the cultural traditions and rituals surrounding pregnancy in her community, which provide her with additional support during this period. **Ecological Factors**: She lives in a safe, green neighbourhood, which helps her relax and enjoy daily walks, contributing to both her Physical and Mental Well-Being
**Key Attributes of PMWB**	**Emotional Well-Being:** Jane experiences a range of positive emotions, including excitement and contentment about the impending arrival of her baby. She practices mindfulness and relaxation techniques learned in her prenatal classes. Jane not only experiences joy but also maintains mood stability, which helps her cope with the daily changes and challenges of pregnancy **Psychological Well-Being:** Jane is engaged in personal growth as she prepares for motherhood. She feels a strong sense of purpose in her new role and has developed a positive self-identity as a mother-to-be. She accepts the challenges of pregnancy and adapts by using effective coping strategies **Social Well-Being:** Jane feels socially connected and supported, both by her immediate family and the broader community. Her relationships are a source of strength, and she actively participates in social networks that provide both emotional and practical support
**Consequences**	**Mother-Infant Bonding:** Jane feels increasingly attached to her baby and looks forward to building a strong bond. Her emotional stability and positive outlook contribute to fostering her baby’s cognitive, emotional, and social development **Impact on Maternal and Infant Health:** Through regular health check-ups and effective coping strategies, Jane reduces her risk of postpartum depression, resulting in improved health outcomes for both her and her baby **Healthcare Cost Reduction:** Jane’s stable physical and Mental Well-Being decreases the need for intensive medical care, leading to more efficient use of healthcare resources **Stable Family Relationships:** Jane’s stable relationship with her partner and the support from her family create a harmonious family dynamic, benefiting both the parents and the child **Long-Term Resilience:** The resilience Jane develops during her pregnancy will help her navigate future challenges as a mother while laying the foundation for her child’s long-term health and Mental Well-Being
	**Borderline Case of PMWB**
**Context**	Laura is a 28-year-old woman in her second trimester of pregnancy. Although she has a supportive partner, Laura is dealing with high levels of work-related stress. She has a history of anxiety, which has resurfaced during her pregnancy. Laura's physical health is generally good, but her mental health is a concern
**Antecedents**	**Social Support** Laura’s partner is supportive, but they both work long hours, limiting their time together. Due to her limited social interactions outside of work, Laura lacks the broader community support that could help alleviate her stress **Economic Stability** Laura and her partner are financially stable, but her demanding job contributes to her stress and anxiety **Access to Healthcare** Laura has regular prenatal visits but feels that her mental health concerns are not fully addressed by her healthcare providers **Education and Awareness** Laura attended prenatal classes, but her anxiety prevents her from fully engaging with the material **Physical Health** Laura’s physical health is generally good; she maintains a balanced diet and does not have any chronic conditions. However, her high stress levels occasionally interfere with her ability to sleep and stay physically active **Psychosocial Resilience** Laura struggles with psychological resilience due to her history of anxiety. Her limited coping mechanisms make it difficult for her to adapt to the stressors of pregnancy, leaving her feeling overwhelmed and less confident in her ability to navigate this period effectively **Cultural and Environmental Factors** Laura’s urban living environment, characterized by limited access to green spaces, further exacerbates her stress levels, as she lacks opportunities for relaxation and physical activity in nature
**Key Attributes of PMWB**	**Emotional Well-Being**: Laura struggles with maintaining positive emotions due to her anxiety and work stress. She experiences mood swings and feelings of inadequacy about her ability to be a good mother. Laura's chronic fatigue and lack of physical activity further diminish her ability to cope with the daily stresses of pregnancy, negatively impacting her Mental Well-Being **Psychological Well-Being**: Laura’s anxiety interferes with her sense of personal growth and self-acceptance. She often feels overwhelmed and doubts her coping strategies. Despite attending prenatal classes, her anxiety prevents her from fully embracing her new role as a mother, limiting her sense of purpose and fulfilment **Social Well-Being**: Although Laura has some social support, her work schedule and anxiety limit her ability to connect with others and participate in social networks
**Consequences**	**Impact on Maternal and Infant Health** Laura’s mental health concerns, if left unaddressed, increase her risk of postpartum depression. Although her physical health is stable, her elevated stress levels and limited resilience may lead to complications, affecting both her and her baby’s overall health outcomes **Healthcare Cost Reduction** Laura’s persistent mental health challenges and high stress could lead to increased reliance on healthcare services, including potential interventions for mental health conditions postpartum. Without targeted PMWB interventions, her situation may result in higher healthcare costs **Stable Family Relationships** While Laura has a supportive partner, the limited time they spend together due to demanding jobs puts a strain on their relationship. The lack of adequate family time and her high stress levels could create relational tensions, potentially impacting the supportive environment needed for her and her baby **Long-Term Resilience** Laura’s low psychological resilience and ongoing anxiety pose challenges for her ability to adapt to motherhood effectively. Without strengthened coping mechanisms and targeted mental health support, her capacity to develop long-term resilience for both herself and her child may remain limited
	**Related Case of PMWB**
**Context**	**Emily** is a 32-year-old woman who recently gave birth to her second child. During her pregnancy, she faced several challenges, including the illness of her elderly mother and managing the care of her first child. Despite these challenges, Emily has been able to maintain a generally positive outlook
**Antecedents**	**Social Support** Emily has a supportive spouse and extended family who help with childcare and household responsibilities **Economic Stability** Emily and her partner are financially comfortable, allowing her to take maternity leave without financial strain. **Access to Healthcare** Emily received consistent prenatal care, and her healthcare providers were attentive to both her physical and mental health needs **Education and Awareness** Emily is well-informed about the importance of mental health during the perinatal period, having attended support groups and prenatal classes **Physical Health** Emily maintains good physical health, supported by balanced nutrition and regular check-ups during and after pregnancy. However, the physical demands of caring for her newborn and her elderly mother occasionally leave her feeling fatigued, which she manages with rest and practical support from her family **Psychosocial Resilience** Emily demonstrates strong psychosocial resilience by employing various coping strategies to manage stress. She regularly practices mindfulness exercises, which help her stay calm and focused during challenging moments. Her ability to seek and accept help from her partner and family further enhances her capacity to navigate the dual responsibilities of motherhood and caregiving **Cultural and Environmental Factors** Emily’s close-knit cultural community provides her with a strong sense of belonging, which plays a crucial role in supporting her Mental Well-Being during the postpartum period
**Key Attributes of PMWB**	**Emotional Well-Being:** Emily experiences a mix of positive emotions, such as love and joy, but also occasional feelings of sadness and stress due to her caregiving responsibilities. **Psychological Well-Being:** Emily shows resilience and personal growth as she navigates the challenges of caring for a newborn and her ailing mother. She maintains a sense of purpose in her role as a mother **Social Well-Being:** Emily’s strong social network plays a crucial role in maintaining her Mental Well-Being. Her partner takes an active role in parenting and provides emotional support during moments of overwhelm. Furthermore, Emily maintains regular contact with her neighbours and members of her cultural community, who encourage her and offer practical help, such as babysitting services. These social connections provide Emily with a sense of belonging and enable her to maintain a positive outlook, even in challenging times
**Consequences**	**Mother-Infant Bonding and Child Development** Emily’s strong Mental Well-Being enables her to be emotionally available for her children, fostering a secure attachment and positive parent–child relationships. Her proactive involvement in her newborn’s care and the emotional bond she shares with her older child create a nurturing environment that supports her children’s emotional and cognitive development **Impact on Maternal and Infant Health** Emily’s coping strategies and ability to manage stress effectively protect her from developing postpartum depression. This contributes to her overall Well-Being and ensures that she remains physically and mentally healthy, benefiting both her and her newborn. By addressing her mental health needs, Emily avoids potential complications that could impact her caregiving capacity **Healthcare Cost Reduction** Emily’s proactive approach to maintaining her mental and Physical Well-Being reduces the likelihood of requiring intensive healthcare interventions. Her ability to prevent mental health crises contributes to more efficient use of healthcare resources, minimizing costs for both her family and the healthcare system **Stable Family Relationships** Emily’s strong relationship with her spouse and her extended family fosters a supportive and harmonious environment. This stability allows her to balance her caregiving responsibilities and ensures that her children grow up in a positive and emotionally secure family setting. Her ability to manage stress strengthens her relationships and reduces the risk of familial tension **Long-Term Resilience** Emily’s ability to adapt to the demands of motherhood, coupled with her coping strategies and strong social network, builds long-term resilience. This not only benefits her own Mental Well-Being but also sets a positive example for her children, helping them develop emotional adaptability and resilience over time
	**Contrasting Case of PMWB**
**Context**	**Sophie** is a 25-year-old woman in her second trimester of pregnancy. Sophie is a single mum who doesn't have a lot of friends and is having a lot of money problems. Her job pays little and doesn't give her regular pregnancy care, and her hours aren't consistent. Sophie experienced sadness in the past, which worsened during her pregnancy
**Antecedents**	**Social Support** Sophie lacks a strong support system. She has minimal contact with her family, and her friends are not available to provide consistent help **Economic Stability** Sophie is financially unstable, struggling to afford basic necessities, including healthy food and prenatal care **Access to Healthcare** Due to her financial situation and irregular work hours, Sophie has missed several prenatal appointments and does not have consistent access to healthcare services **Education and Awareness** Sophie has not attended prenatal classes and has limited knowledge about the physical and emotional changes during pregnancy **Physical Health** Sophie’s poor physical health is characterized by inadequate nutrition and persistent fatigue. These issues, exacerbated by financial instability, negatively affect her ability to manage the physical demands of pregnancy **Psychosocial Resilience** Due to her history of depression, Sophie lacks effective coping mechanisms, which makes it challenging for her to handle the stressors of pregnancy. This exacerbates her feelings of hopelessness and emotional detachment **Cultural and Environmental Factors** Living in an overcrowded and noisy environment with limited access to green spaces further reduces Sophie’s ability to find moments of peace, increasing her overall stress levels
**Key Attributes of PMWB**	**Emotional Well-Being:**Sophie experiences frequent negative emotions, including sadness, fear, and hopelessness. She has difficulty finding joy in her pregnancy and often feels overwhelmed by her circumstances. Sophie feels isolated and disconnected, with little to no social support. Her limited access to prenatal healthcare prevents her from receiving timely support and interventions, further exacerbating her feelings of stress and anxiety during her pregnancy. As a result, she struggles to find moments of emotional relief. **Psychological Well-Being:** Sophie struggles with low self-esteem, a lack of purpose, and persistent feelings of failure. Her history of depression exacerbates these issues, making it difficult for her to cope with the challenges of pregnancy. Sophie's limited access to prenatal healthcare services prevents her from receiving essential guidance and support, which could have alleviated some of her anxiety and stress during pregnancy. **Social Well-Being:** Sophie feels isolated and disconnected, with little to no social support. Her lack of social networks contributes to her feelings of loneliness and stress
**Consequences**	**Mother-Infant Bonding and Child Development** Sophie’s persistent sadness, emotional detachment, and lack of joy during pregnancy prevent her from forming a meaningful connection with her baby. This lack of connection may lead to attachment issues in the early stages of her child’s life, potentially affecting the child’s emotional development and Mental Well-Being **Impact on Maternal and Infant Health** Sophie’s poor physical health, including inadequate nutrition and persistent fatigue, exacerbates her feelings of helplessness and deepens her emotional distress. Her pre-existing mental health issues, coupled with financial stress and lack of access to healthcare, place her at a high risk of postpartum depression, which could have lasting effects on both her and her baby’s health **Healthcare Cost Reduction** The absence of adequate prenatal care and her unmet mental health needs may result in increased reliance on healthcare services after childbirth. Without preventive interventions, Sophie’s situation could lead to higher healthcare costs in the long term **Stable Family Relationships** Sophie’s lack of social support and isolation exacerbate her feelings of loneliness and stress. This absence of a supportive partner or family dynamic further reduces her ability to create a stable and nurturing environment for her child **Long-Term Resilience** Without interventions to build her resilience and coping skills, Sophie is likely to continue struggling with depression beyond the perinatal period. This may have a long-term impact on her mental health and reduce her ability to foster a stable and supportive environment for her child, potentially affecting the child’s emotional and Psychological Well-Being

This table presents four case analyses illustrating different manifestations of Perinatal Mental Well-Being (PMWB). The Model Case represents an optimal scenario with strong antecedents and positive consequences, while the Borderline Case demonstrates moderate challenges affecting PMWB. The Related Case highlights resilience despite external stressors, and the Contrasting Case shows significant risk factors that negatively impact PMWB. Each case includes contextual information, antecedents, key attributes, and consequences to illustrate the complexity of PMWB in different maternal experiences

### Analysis

This case illustrates a model case with core attributes, antecedents, and consequences of PMWB within the context of a real-life scenario, elucidating the role of social support, economic security, and access to health services. This has significant implications for a holistic conceptualisation of perinatal maternal mental health, demonstrating how optimal support systems can foster positive outcomes for both mothers and children.

### Step 6: identifying borderline, related and contrasting case

#### Borderline and related cases

The borderline case of Laura illustrates a situation in which some aspects of PMWB are present, but defining elements, particularly in Emotional Well-Being, are limited due to a continuous state of anxiety. In contrast, the related case highlights how Emily's narrative demonstrates that strong social support and resilience contribute to maintaining PMWB, even when facing significant challenges.

### Analysis

These cases illustrate the complexity of PMWB, where varying combinations of social support, psychological resilience, and economic stability either sustain or exacerbate positive or negative outcomes for mothers and children. The borderline and related cases are used in this analysis to thoroughly assess PMWB and identify its most relevant antecedents and consequences.

### Contrasting case

This case highlights the absence of supportive antecedents and its effects on PMWB. It demonstrates how the lack of social support, economic stability, and medical care can severely compromise PMWB during the perinatal period.

### Analysis

While the model and related cases illustrate circumstances where PMWB attributes are positively expressed, the contrasting case focuses on situations where these attributes are absent or negatively expressed. This underscores the critical role of supportive antecedents, such as social support and economic stability, in achieving PMWB during the perinatal period. Sophie’s case illustrates how the absence of these antecedents significantly compromises her PMWB. This lack not only affects her PMWB but also hinders her child’s development and disrupts mother-infant attachment. Sophie’s experience highlights the necessity of social support, economic stability, and access to medical care as antecedents for achieving PMWB and preventing adverse outcomes.

A comprehensive summary of the Model, Borderline, Related, and Contrasting Cases of Perinatal Mental Well-Being (PMWB) is provided in Table 4. This table presents key contextual details, antecedents, defining attributes, and consequences for each case. By outlining these cases, the table offers a structured comparison of how different factors influence PMWB outcomes in the perinatal period. For a detailed overview, see Table 4 at the end of this document.

### Interconnection with other dimensions of well-being

PMWB is deeply interconnected with other dimensions of the broader Well-Being framework, including Physical, Spiritual, Economic, and Ecological Well-Being. These dimensions interact dynamically, influencing both the mother’s overall health and her PMWB during the perinatal period Table 5.

**Table 5:** Standard reporting framework for concept analysis according to walker and avant

Step	Title	Description
Step 1	Select a concept	Choose a concept that is relevant to your area of study and lacks conceptual clarity
Step 2	Determine the aims or purposes of analysis	Define why the concept analysis is being performed and what you hope to achieve
Step 3	Identify all uses of the concept	Review the literature to find all uses and definitions of the concept across disciplines
Step 4	Determine the defining attributes	Identify core characteristics of the concept that appear repeatedly across sources
Step 5	Construct a model case	Develop an example that includes all defining attributes of the concept
Step 6	Construct borderline, related, contrary, invented, and illegitimate cases	Create examples that help clarify what the concept is and is not
Step 7	Identify antecedents and consequences	Determine what precedes (antecedents) and results from (consequences) the concept
Step 8	Define empirical referents	Specify how the concept can be measured or recognized in practice

For instance, Laura’s borderline case demonstrates how limited access to green spaces (Ecological WB) and work-related stress due to financial instability (Economic WB) exacerbate her anxiety, reducing her Emotional Well-Being. Similarly, Sophie’s contrasting case highlights how poor nutrition and inadequate healthcare access (Physical WB) further diminish her ability to maintain resilience and Psychological Well-Being. In contrast, Emily’s related case illustrates how a supportive cultural environment (Ecological WB) and strong social networks enhance her resilience and enable her to maintain Emotional and Psychological Well-Being, despite significant caregiving challenges.

These cases collectively underscore the importance of addressing interconnected dimensions of Well-Being. By ensuring access to stable resources, supportive environments, and opportunities for meaningful experiences, mothers can achieve a more balanced and holistic state of PMWB.

### Step 7: identify antecedents and consequences

Walker and Avant (2011) assert that concept analysis often overlooks or undervalues the establishment of antecedents and outcomes [38]. However, a precise identification can be beneficial in understanding the socio-cultural contexts of the concept's use and its deeper resonances. Antecedents are the conditions necessary for the concept to occur, while consequences are the outcomes of its occurrence.

### Major antecedents of PMWB

According to the related literature review, the major antecedents to PMWB are social support [67] economic stability [68], access to healthcare [69], education and awareness [70], physical health [71], and psychosocial resilience [72].

#### Social support

During the perinatal period, adequate support from family, friends, and providers plays a crucial role in reducing stress and providing both emotional and practical assistance [67].

#### Economic stability

Financial stability and access to essential resources contribute to higher levels of PMWB by reducing financial pressures and improving access to both healthcare and basic needs [68].

#### Access to healthcare

Access to regular and available prenatal and postnatal care significantly contributes to early detection and adequate management of disorders, resulting in a better outcome [69].

#### Education and awareness

Recognition of perinatal mental disorders, supported by educational resources, can also enable individual women to seek care and engage in healthier behaviours both during pregnancy and in the postpartum period [70].

#### Physical health

The overall physical health status of the mother is another critical determinant of performance, which includes a lack of chronic conditions and good nutritional status [71].

#### Psychosocial resilience

Psychological resilience, effective coping mechanisms, and a positive outlook are critical psychosocial antecedents that enable women to navigate the perinatal period with adaptive capacity and resolve [72].

#### Cultural and environmental factors

In addition to the core antecedents identified, cultural and environmental factors, such as societal expectations and safe living conditions, also play a critical role in shaping PMWB. These factors can influence a mother’s stress levels, access to social support, and mental health resilience during the perinatal period [73, 74].

### Key Consequences of PMWB

#### Mother-infant bonding and child development

A positive PMWB boosts the psychological attachment of mothers to their infants, which helps them support their child’s social and cognitive development [1]. Good maternal mental health enhances childhood development characteristics like language, cognitive, and emotional performance [75, 76].

#### Impact on maternal and infant health

PMWB helps improve perinatal mental health and reduces the risk of mental health problems. [1, 59]. Since PMWB interventions decrease the risk of postnatal depression, it results in better health for the mother and child with less need for healthcare interventions [59].

#### Healthcare cost reduction

Higher regard for PMWB can help reduce healthcare costs by lowering the incidence and impact of mental health issues, leading to a more efficient use of available resources [77].

#### Stable family relationships

PMWB strengthens family relationships, particularly between partners, by fostering a stable and supportive environment that benefits both the parents and the child’s development [15, 28]. Strained relationships, however, raise health risks for both mother and child, highlighting the need for support interventions involving both partners [1, 59].

#### Long-term resilience

The sustained PMWB allows for resilience both in the mother and child; hence, this supports health and PMWB the long term. Overall, PMWB benefits maternal and child health for broader public health by emphasizing its societal importance in general [28].

### Step 8. Defining the empirical referents

Establishing empirical references is necessary to adequately conceptualise the identification and measurement of the PMWB concept. Research and clinical practice can demonstrate and measure the concept of PMWB using empirical referents [38]. Empirical referents allow abstract ideas like PMWB to be operationalised as observable and measurable parts. This step is crucial for verifying the accuracy of PMWB measures and establishing a strong basis for creating interventions that support maternal mental health during the perinatal period [38].

#### Empirical tools for measuring PMWB

The"Capture My Mood"(CMM) enables women to self-monitor PMWB by tracking their emotional and psychological states in real-time throughout the perinatal period [41]. The CMM scale enables self-assessment for early detection of mental health problems, facilitating timely support and interventions by healthcare providers. The CMM scale has shown a strong correlation with the Warwick-Edinburgh Mental Well-Being Scale (WEMWBS), a validated tool for assessing General Mental Well-Being across all life stages [78]. This correlation validates the reliability of the CMM scale and allows PMWB estimates to be benchmarked against established tools like the WEMWBS.

Building on the insights from the CMM scale, the Well-Being in Pregnancy (WiP) questionnaire has also been validated as a key tool for PMWB assessment [57]. Researchers refined the WiP questionnaire through cognitive interviews and psychometric validation, which ensures its relevance and accuracy in assessing pregnant women's PMWB [57]. It is a multifunctioning tool, combining GWB items and specific questions relevant to the challenges experienced by women in pregnancy, making it versatile for both clinical and research applications [57].

#### Practical applications

Women who used the CMM tool reported it to be easy, user-friendly, and especially useful at an early postnatal period [41]. The combined use of the CMM scale and WiP questionnaire, alongside standard measures like WEMWBS, facilitates a multi-dimensional approach to PMWB. These empirical referents are essential in providing timely support to women during the perinatal period, ultimately enhancing maternal and infant health outcomes.

## Discussion

The concept analysis of PMWB provides a deeper view of the multidimensional nature of maternal mental health throughout the perinatal period and identifies Emotional, Psychological, and Social WB as core components. Each core dimension contributes uniquely to the overall framework of PMWB, yet they are deeply interconnected.

Emotional Well-Being encompasses the ability to experience positive feelings, maintain mood stability, and derive life satisfaction and happiness throughout the perinatal period. This dimension is particularly influenced by hormonal changes, physical health, and the presence of supportive relationships, which can either buffer or exacerbate emotional challenges.

Psychological Well-Being reflects a deeper sense of personal growth, autonomy, self-acceptance, and purpose in life. These qualities are essential for women as they navigate the significant role transitions and identity shifts associated with motherhood. Developing psychological resilience and fostering a strong sense of mastery can help women adapt more effectively to the demands of the perinatal period.

Social Well-Being encompasses the mother’s sense of social coherence, integration, and connectedness within her community. It also involves feelings of meaningful contribution and belonging in her social environment. Social support networks, including family, friends, and healthcare providers, are vital for reinforcing this dimension, as they help women feel valued and reduce feelings of isolation or stress.

Together, these three core components form the foundation of the Mental dimension in the perinatal period, with changes in one domain often influencing the others. For instance, a lack of social support can negatively impact emotional stability and undermine a woman’s sense of self-efficacy and purpose. By addressing these dimensions holistically, PMWB can be more effectively supported and optimized throughout the perinatal period.

Considering these Mental dimensions, the analysis underlines that PMWB cannot be viewed outside broader conceptualisations of WB, including Physical, Spiritual, Economic, and Ecological factors. The interrelationship between various dimensions highlights that changes in one domain can significantly influence outcomes in others. These findings align with previous studies that established the importance of a holistic approach in maternal health practices [15, 56]. It is also crucial to recognize that PMWB is a dynamic concept characterized by subjective positive self-evaluation and fluctuations throughout the perinatal period. For example, hormonal changes and physical discomfort may vary across different stages of pregnancy, while psychological factors like fear of childbirth, role transitions, and evolving social support systems can dynamically influence a woman's mental state [6, 66, 69, 79] Additionally, PMWB is a dynamic concept, influenced by the cultural and social environment in which women live and experience their Mental Well-Being throughout the perinatal period [67, 73, 74].

### Implications for midwifery and maternal healthcare

One important finding of this study is the critical role midwives and other healthcare providers play in identifying and supporting PMWB. Midwives hold an important position in offering psychosocial, integrated support through routine care because they can often remain in close, trusted contact with women throughout the perinatal period [28]. At the same time, Howard et al. (2014) emphasize the critical importance of integrating mental health support into perinatal care, which includes enhancing midwifery education to improve support for PMWB [1].

In addition to using screening tools to identify PMWB concerns, midwives can play a proactive role in enhancing maternal resilience, which has been shown to buffer stress and improve coping mechanisms during the perinatal period. Strategies such as promoting stress management techniques, facilitating access to peer support groups, and encouraging self-care practices can help build maternal resilience [80]. For instance, interventions such as mindfulness-based stress reduction programs, psychoeducation sessions, and structured peer support initiatives have been identified as effective approaches for enhancing maternal resilience and reducing stress during the perinatal period [81, 82]. This aligns with findings from Van Haeken et al. (2023), who developed a systematic resilience-enhancing intervention framework informed by the Behaviour Change Wheel [83]. This approach emphasizes the importance of measurement in guiding tailored interventions aimed at enhancing resilience during the perinatal period [83]. Moreover, the findings of Van Haeken et al. (2020) highlight that strengthening resilience through attributes such as social support, self-efficacy, self-esteem, a sense of mastery and positive personality traits can significantly enhance PMWB [84]. These attributes align closely with the core dimensions of PMWB identified in this study: Emotional, Psychological, and Social Well-Being. Social support contributes to Social Well-Being by fostering connectedness and reducing isolation, while self-efficacy and self-esteem strengthen Psychological Well-Being by promoting autonomy, self-acceptance, and personal growth. Additionally, optimism and other positive personality traits help sustain Emotional Well-Being by enhancing emotional stability and fostering positive feelings [84].

By focusing on these attributes, midwives can adopt a resilience-enhancing approach, reinforcing both Psychological Emotional and Social Well-Being in women during the perinatal period. These strategies not only mitigate stress but also foster a sense of personal growth and adaptation, underscoring the interconnected and holistic nature of PMWB [84].

### Challenges in defining and measuring PMWB

Challenges in Defining and Measuring PMWB Empirical referents such as the Capture My Mood (CMM) scale and the Well-Being in Pregnancy (WiP) questionnaire provide a foundation that enables the quantification of PMWB. However, challenges remain, particularly toward arriving at universally valid and reliable measures across diverse cultural and socioeconomic contexts [41]. Women using the CMM scale reported it as easy to use and particularly helpful in the early days after birth when mental health support is most needed [78]. The high correlation between the CMM scale and the Warwick-Edinburgh Mental Well-Being Scale (WEMWBS) validates the reliability of the CMM scale, allowing PMWB estimates to be benchmarked against an established and widely recognized tool for assessing general Mental Well-Being [78, 85].

The current validation studies for the CMM and WiP tools are primarily based on homogeneous or Western populations, limiting their generalizability and their applicability in culturally diverse settings where understandings of mental health and Well-Being may differ [41, 57, 73]. Therefore, these tools need further research to establish their validity across various cultural and socio-economic settings and the ability of their accurate linking with the subtlety of PMWB [69].

Furthermore, PMWB deeply embedded in cultural understandings of motherhood, emotional expression, and social support. Studies have shown that cultural norms influence not only how symptoms of distress are experienced and expressed, but also how they are recognised and addressed by health professionals [7, 73, 86]. For instance, perceptions of what constitutes “Well-Being” may vary significantly across populations, with some cultures emphasising emotional restraint or collective responsibility over individual psychological flourishing [87]. As a result, tools developed and validated in predominantly Western contexts may fail to capture culturally nuanced manifestations of PMWB, potentially leading to under-identification or misclassification of mental health needs. This underscores the importance of culturally sensitive frameworks and the cross-cultural validation of measurement instruments in future research [7, 73, 86, 87].

### Critical reflections

This analysis reveals that while Emotional, Psychological, and Social are well represented in maternal health frameworks, dimensions like Spiritual, Economic and Ecological Well-Being are often overlooked. For instance, Social Coherence, which reflects the sense of community and mutual trust, was explicitly mentioned in only two studies, while Ecological Well-Being had no explicit mention. These findings suggest that these dimensions are often overlooked in the literature, potentially limiting the holistic understanding of PMWB. Addressing these gaps in future research is crucial to ensure a comprehensive approach to maternal mental health.

Additionally, since limited PMWB-specific studies are currently available, this review had to draw insights from studies focused on GWB. While such studies had useful insights, it also meant there was variability in scope and focus, complicating any direct comparisons and generalizability of findings to the perinatal period specifically. However, given the ongoing evolution of PMWB concepts, future research must clarify PMWB's unique attributes to distinguish it from broader mental health constructs [12].

Finally, there is the assumption in much of the literature that PMWB can be universally defined and measured, when in fact it is most likely that different perinatal populations experience and interpret PMWB uniquely. For example, the economic stability component of PMWB may be experienced in low-resource settings quite differently compared to high-income contexts, where different aspects of PMWB might be weighted more by women due to varying levels of resources and social support [1]. This analysis underscores that a comprehensive understanding of PMWB cannot be separated from the cultural context in which women live. Without acknowledging cultural diversity, important aspects of PMWB, such as traditional postpartum practices or the stigma surrounding mental health; may remain overlooked [67, 73, 74].

### Future directions

Future research should prioritize the inclusion of underrepresented dimensions such as Spiritual, Economic and Ecological Well-Being. Exploring how these dimensions interact with core components like Emotional, Psychological, and Social Well-Being could provide a fuller understanding of PMWB. Moreover, investigating these dimensions would help address the current gaps in the literature and ensure a more comprehensive and inclusive approach to maternal mental health.

Future research should be directed to the construction of valid and culturally sensitive tools and methods to accommodate the diversity of perinatal populations [57, 73, 88]. Concomitantly, advancing the field of digital health, new modalities such as telemedicine and mobile applications offer novel opportunities for real-time mood tracking, thus enabling continuous support for PMWB. These can ensure that mental health challenges, when arising, will be timely addressed, through instantaneous feedback and, thereby, early intervention along with other advantages [89]. Digital interventions, in particular, in low-resource settings could contribute to the closing of critical gaps in maternal mental health care, where conventional services are usually limited because of resource constraints [28, 77]​. By incorporating digital health solutions, maternal mental health support can become more accessible, responsive, and tailored to individual needs, ultimately enhancing PMWB outcomes on a broader scale.

These findings have important consequences for clinical practice. The key attributes and antecedents of PMWB identified in this analysis can guide the development of more holistic screening methods. These should focus not only on risks, but also on strengths. Midwives and other maternity care providers could benefit from practical tools and training. These should cover both protective and promotive aspects of Mental Well-Being.

The focus on conception as a starting point also underlines the need for earlier psychosocial support. Ideally, this support begins even before pregnancy, or at least in the very early stages. PMWB is shaped by both cultural and personal factors. Therefore, clinical interventions must be flexible and culturally sensitive. By applying these insights in daily care, early detection may improve. At the same time, maternal support systems can be strengthened, leading to better long-term outcomes for mothers and their children.

This study consequently provides a clearer conceptualisation of PMWB, recognizing its importance in both maternal and child health. Application of this framework to clinical service will thus ensure better maternal mental health outcomes, ultimately benefiting families and communities. The adequately resourced holistic approach to PMWB and practical assessment greatly holds promise to transform maternal mental health care in the perinatal period.

## Strengths and limitations

It represents the first review of concepts related to PMWB during pregnancy, childbirth, and up to one year after birth. In this sense, it makes a new contribution to research on maternal mental health. This analysis puts forward Emotional, Psychological, and Social Well-Being as core dimensions, integrating broader factors such as Physical, Spiritual, Economic, and Ecological Well-Being for a comprehensive and holistic framework in understanding PMWB. This constitutes a fundamental strength since the basis of further research and practical interventions is constituted thereby.

A critical limitation is the reliance on studies focused on general WB or MWB rather than specifically on PMWB. While these studies added value by illuminating the mental aspects of PMWB, they introduced variability in scope and content, complicating direct comparisons and highlighting the need for research directly addressing PMWB.

The diverse composition of the research team including mothers, fathers, midwives, nurses, and a psychologist is at once considered both a factor of their strength and potential limitations. While such personal and professional experiences enriched the analysis by bringing multiple perspectives, they may equally introduce interpretive bias. A similar analysis conducted by another, or more diverse research team could consider other perspectives and provide validation of the findings presented here.

The abstract nature of this analysis also places limitations in terms of applicability to empirical practice. This provides a framework for tool and intervention development based on further validation that can be extrapolated to measurable and actionable frameworks, particularly across different cultural and socioeconomic statuses.

## Conclusion

PMWB is a dynamic and multifaceted concept that encompasses a subjective positive self-evaluation across three primary dimensions: Emotional, Psychological, and Social WB. Emotional WB includes elements such as positive emotions, life satisfaction, mood stability and happiness. Psychological WB encompasses personal growth, autonomy, purpose in life, self-acceptance, and self-esteem. Social WB involves social coherence, social integration, connectedness, and social contribution.

This analysis highlights the interconnectedness between PMWB and broader dimensions of General Well-Being, including Physical, Spiritual, Economic, and Ecological aspects. Recognizing the reciprocal influence among these factors is essential, as a woman's PMWB is likely to evolve dynamically throughout the perinatal period in response to the ongoing development of parenthood.

Based on the attributes identified in this concept analysis, we propose the development of a culturally sensitive scale for women to self-monitor their PMWB. This scale is intended to alert women to changes in their mental status, facilitating timely support and interventions that can enhance maternal mental health throughout the perinatal period. Future research should focus on validating such a scale across diverse populations and developing targeted interventions that address the multidimensional nature of PMWB. This framework not only enhances maternal mental health care but also contributes to improved health outcomes for families and communities by promoting holistic and dynamic approaches to perinatal care and recognizing mental health as a core element of overall maternal Well-Being.

## Supplementary Information

Supplementary Material 1.

Supplementary Material 2.

## Data Availability

All data generated or analysed during this study are included in this published article and its supplementary files.

## Abbreviations

PMWB: Perinatal Mental Well-Being
PWB: Perinatal Well-Being
GWB: General Well-Being
MWB: Mental Well-Being
WB: Well-Being
