# Supplementary material for: Exploring perinatal mental well-being: a concept analysis from conception to one year postpartum
Source: BMC Pregnancy Childbirth. 2025 Oct 10;25:1068. doi: 10.1186/s12884-025-08015-y (PMC12512377; doi:10.1186/s12884-025-08015-y)
Supplement: Supplementary file 1 — Supplementary Material 1. [file 12884_2025_8015_MOESM1_ESM.docx]

| Article & Auteur & Country of study | Design &  Type of Well-Being | | Aim of the study & Population | Key Findings: Definitions and Concepts  Key findings or theoretical Insights related to PMWB |  | |
| --- | --- | --- | --- | --- | --- | --- |
| 1.Capture my mood: a feasibility study to develop a visual scale for women to self-monitor their mental well-being following birth (McGinley et al.,2017) (1).  Australia | Feasibility Study and Mixed-Methods  **Perinatal Mental Well-Being** | **Aim** was to explore the practicality of developing a new tool, the "Capture My Mood" (CMM) visual scale, and to assess how users experience the tool.  The study focuses specifically on **self-monitoring of Mental Well-Being** in women postpartum, emphasizing emotional and psychological aspects of well-being.  **Population**  N=30 postpartum women | | The CMM tool uses a visual scale with **five descriptors** aligned with the **WEMWBS**:   - **Connected:** focus on satisfying interpersonal relationship - **Confident:** positive functioning - **Cheerful:** positive affect - **Contented:** pleasure and satisfaction - **Capable:** self-fulfilment and purpose |  | |
| 2.Preliminary evaluation of the Well-being in Pregnancy (WiP) questionnaire (Alderdice et al.,2017) (2).  United Kingdom | Feasibility Study and Mixed-Methods  **Perinatal Well-Being** | | **Aim of the Study**  The aim was to develop and evaluate the Well-Being in Pregnancy (WiP) questionnaire to measure both positive and negative emotions during pregnancy. It aims to capture a holistic view of a woman's emotional state during pregnancy.  **Population**: Pregnant women across various stages of pregnancy. | **The concept of Well-Being** is **multi-faceted by encompassing** both positive and negative emotions and satisfaction with life. Measuring both positive and negative thoughts and emotions is highly relevant in maternity care that aims to optimize a ''woman's experience of pregnancy and childbirth’, focusing on positive aspects of health and Well-Being, not just the prevention of ill health.  **Measure**: The questionnaire is structured around 12 pregnancy-specific questions and identifies two main factors:   - Positive experiences of pregnancy, which relate to positive affect and overall satisfaction. - Concerns about health and pregnancy outcomes, capturing negative affect and worries associated with the pregnancy experience. |  | |
| 3.Well-Being in high-risk pregnancy: an integrative review (Mirzakhani et al., 2020) (3).  Iran | Integrative Review  **Perinatal Well-Being** | | **Aim**: To explore the concept of Well-Being in the context of high-risk pregnancies (HRP) and to identify the factors that influence it.  **Population**  Focused on women experiencing high-risk pregnancies. | Well-Being in high-risk pregnancy is a **multidimensional**, complex and abstract subjective concept and a cognitive and emotional self-evaluation of one’s own life in HRP. **Its four main dimensions:**   - **Physical** - **Mental-Emotional Well-Being** - **Social Well-Being** - **Spiritual Well-Being** |  | |
| 4. Conceptualising women's perinatal well-being: A systematic review of theoretical discussions (Wadephul et al.,2020) (4).  United Kingdom | Systematic Review  **Perinatal Well-Being** | | **Aim**  To explore theoretical discussions on the concept of perinatal well-being.  The study reviews existing literature to identify elements, dimensions, and gaps in the understanding of perinatal Well-Being.  Focuses on synthesizing a comprehensive framework that goes beyond physical and psychological aspects, to include social and emotional dimensions**.**  **Population**  N= 318 pregnant women​. | **The concept of Perinatal Well-Being** is a **complex, multidimensional construct.** Three themes were developed:  **(1)** The importance of a number of general domains  of women's lives and domains specific to the perinatal period,  **(2**) **Well-Being as a subjective and individual experience** with physical/embodied, affective, and psychological/cognitive aspects  **(3)** **The dynamic nature of Well-Being.** |  | |
| 5. What is perinatal well-being? A concept analysis and review of the literature (Allan et al.,2013) (5).  United Kingdom | Concept analysis and Literature review  **Perinatal Well-Being** | | **Aim**  Systematic literature review  **Population**  Women in the perinatal period | **The apparent attributes of the apparent attributes** of **‘Perinatal Well-Being’**:  **(a)The time period** ranging from before and after childbirth  **(b)** **multidimensional** elements, which include physical, psychological, social, spiritual, economic, and ecological  **(c)** **subjective cognitive and/or affective self-evaluation of life** |  | |
| 6.Exploring subjective well-being after birth: A qualitative deductive descriptive study (Alderdice & Gargan,2019) (6).  United Kingdom | A qualitative deductive descriptive study of 19 women  **Perinatal Subjective Well-being** | | **Aim**  Explore women's experiences of Well-Being postpartum.  **Population**  **N=19 postpartum women** | **Subjective Well-Being** is a **multidimensional concept** broadly considered to have cognitive (**life satisfaction** and positive functioning) and **emotional** (positive and negative affect) components that are subjective for the individual and relative to social norms and values. |  | |
| 7.Development and Validation of a Mental Well-Being Scale in Singapore (Fen et al.,2013) (7).  Singapore | Scale development & validation study, **Mental Well-being**  **General Mental Well-Being** | | **Aim**  Develop and validate a scale for Mental Well-Being; **N=3400 (multi-ethnic sample)**  **Population**  **N= 3400 adults not in the perinatal period** | **Mental Well-Being** is defined as a state that enables individuals to live life to the fullest, actualize their growth potential, and experience happiness and satisfaction along the way. The concept includes   - **cognitive,** - **emotional,** - **social components**   That reflect the individual’s self-perception and alignment with cultural values. | |  |
| 8.Keyes's model of mental health with personal growth initiative as a parsimonious predictor (Robitschek,2009) (8).  United States | Quantitative Study/ Analytical Cross-Sectional Study with Confirmatory Factor, **Mental Well-Being**  **General Mental Well-Being** | | **Aim**  Test of Keyes’s model on college students  **Population**  College students; **N=467 (244 women, 223 men)** | **Keyes's (2002) multidimensional model of mental health** includes three domains:   - Emotional **Well-Being** - **Psychological Well-Being** - **Social Well-Being**   with 14 subdimensions | |  |
| 9.Towards a ‘patient-centered’ operationalization of the new dynamic concept of health: a mixed-methods study(Huber et al.,2016) (9).  Netherlands | A Mixed-Methods Study  **General Mental Well-Being** | | **Aim**  Operationalize the new dynamic concept of health.  **Population**  **N=2078 (qualitative: 140, quantitative: 1938)**  Healthcare providers, patients, and policymakers | **A new dynamic concept of health**: ‘Health as the ability to adapt and to self-manage, in the face of social, mental and physical challenges of life’.  **Six dimensions of positive Health concept**   - Bodily functions - **Mental functions** - Spiritual/existential dimension - Quality of life Social participation - Daily functioning.   **Mental health and Mental Well-Being** **are one of the cornerstones:**   - Cognitive Functioning. - **Emotional State** - Esteem/Self-respect - Experiencing to be in Charge/Manageability: Sense of control over one’s life. - Self-management - Resilience, SOC (Sense of Coherence): Ability to maintain or regain mental health despite adversity .. | |  |
| 10.Challenges in Defining and Measuring Well-Being and Their Implications for Policy (Huppert, 2013) (10).  United Kingdom | Literature Review and Theoretical Analysis  **General Mental Well-Being** | | **Aim**  Address challenges in defining and measuring Well-Being.  **Population**  **No specific sample** | **Well-being is a multidimensional concept Types of Well-Being in the article:**   - **Cognitive Well-** **Being** - **Emotional Well-Being** - **Psychological Well-Being** - **Social-Well-Being**   **Well-Being** is a **multidimensional concept**, where various components contribute to an overall sense of Well-Being. | |  |
| 11.Review of 99 self-report measures for assessing well-being in adults: exploring dimensions of well-being and developments over time (Linton et al.,2016) (11)  United Kingdom | Review  **General Mental Well-Being** | | **Aim:** Review of 99 self-report measures of Well-Being.  **Population**  **No specific sample** | **A total of 99 measures of** **Well-Being** were included, and 196 dimensions of Well-Being were identified.  Dimensions clustered around **six key thematic** domains:   - Mental-Well-Being - Social Well-Being - Physical Well-Being, Spiritual Well-Being - Activities and functioning, Personal circumstances.   Mental Well-Being includes **cognitive functioning, emotional state, self-management**, and **resilience** as key components​ | |  |
| 12.Mental Well-Being: International Contributions to the Study of Positive Mental Health (Keyes, 2013) (12).  International | Literature Review and Theoretical Analysis:  **Mental Health as a Complete State**  **General Mental Well-Being** | | **Aim:** Explore the dual continua model  **Population**  **No specific sample** | **Explores the Concept of Mental Well-Being**  Mental health is defined as a **complete state**, encompassing both the absence of mental illness and the presence of positive mental health​.  Focuses on three dimensions**:**   - **Emotional,** - **Psychological**, - **Social Well-Being**   emphasizes flourishing as optimal mental health​ | |  |
| 13.A Two-Dimensional Conceptual Framework for Understanding Mental Well-Being (Joshanloo & Weijers, 2019) (13).  International | Research Article  Conceptual Framework Development  Multidimensional scaling analysis, Mental Well-being  **General Mental Well-Being** | | **Aim**: Explore a 2-dimensional structure of Mental Well-Being.  **Population** N=1149 Data from 3 countries (United States, Japan, Iran) | Proposes a two-dimensional framework for understanding. Mental Well-Being, integrating Hedonic and Eudaimonic perspectives.  **Two-Dimensional Framework**  The first dimension: Eudaimonic Well-Being versus Hedonic Well-Being   - **Hedonic Well-Being** - **Eudaimonic Well-Being**   The second dimension: Existential Relatedness vs. Epicurean Independence   - **Existential Relatedness: meaningful** connections with others and the world. - **Epicurean Independence**: autonomy, self-sufficiency, and freedom from negative emotions. | |  |
| 14.Promoting Positive Mental Health and Well-Being: Practice and Policy (Barry, 2017) (14).  International | Policy analysis, **Positive Mental Health**  **General Mental Well-Being-Positive Mental Health** | | **Aim**:  Explore strategies for promoting positive mental health.  **Population:**  **No specific sample** | Mental Well-Being defined as a **multidimensional concept** encompassing **Emotional, Social, and Psychological Well-Being** interventions in community settings to enhance **resilience, social connectedness**, and self-management for positive health outcomes.  **Components of Well-Being**   - **Emotional Well-Being** - **Psychological Well-Being** - **Social Well-Being** | |  |
|  |  | |  |  | |  |
| 15.WHO Definitions of Mental Well-Being  (1946,2004,2012,2018) (15-18).  International | The report is a **policy analysis by the WHO**, evaluating various definitions and approaches to mental health and Well-Being over the years.  **General Mental Well-Being** | | **Aim**: The guidelines re intended to promote public health worldwide.  **Population Global population** | **Key Findings (Concept):**   - **In 1946**, the WHO defined health as a state of complete **Physical, Mental, and Social Well-Being**, emphasizing that health is more than just the absence of disease or infirmity​. - **In 2004 and 2012**, the definitions were expanded to highlight the importance of **Emotional resilience, Psychological Well-Being**, and **social connectedness**. This broader perspective acknowledged the role of mental health in enhancing overall **life satisfaction** and productivity​. - The **2018 update** further emphasized a **holistic approach**, recognizing that positive mental health is integral to overall health. It encouraged health systems to focus on **preventive measures** and the **promotion of Well-Being**, not merely the treatment of illness​.   **Theoretical Insights:**   - The WHO promotes a **multidimensional approach to health**, which integrates **physical, mental, and social dimensions**. This approach stresses that health is not solely defined by the absence of illness but also by the presence of positive health states that contribute to an individual's quality of life​.   This overview offers a detailed look at how WHO conceptualizes health and Well-Being, clearly linking physical, mental, and social health in a comprehensive model. . This aligns with the broader aim of understanding and promoting holistic health frameworks.  **Dimensions and details from WHO Reports**   - **Physical Well-Being**   - Includes physical health free from diseases and infirmities, which is foundational to General Well-Being.   - **Reference**: WHO (1946, 2018). - **Mental Well-Being**   - Emphasizes the capability to realize one's own abilities, manage normal life stresses, work productively, and contribute to the community.   - **Reference**: WHO (2004, 2012, 2018). - **Social Well-Being**   - Highlights the importance of co​participation in social activities as indicators of good health.   - **Reference**: WHO (2004, 2012). - **Integration of Dimensions**   - WHO’s definitions highlight the interconnection between Physical, Mental, and Social Well-Being, underscoring that none of these aspects functions in isolation.   - **Reference**: WHO (1946, 2004, 2012, 2018). - **Holistic Health Approach**   - Encourages a comprehensive view of health that includes not just the absence of disease but also a positive aspect of health in physical, mental, and social dimensions.   - **Reference**: WHO (1946, 2018). - **Functional Aspects** - Defines Well-Being in terms of daily abilities: managing stress, maintaining personal and professional productivity, and the ability to support oneself and the community.   - **Reference**: WHO (2004, 2012, 2018). | |  |
| 16.Mental Health as a Complete State: How the Salutogenic Perspective Completes the Picture(Keyes,2014) (19).  International | Conceptual Analysis and Review  **Positive Mental Health** | | **Aim:**  Explore the dual continua model  **Population**  **Focus on U.S. adults** | Identifies **Emotional, Psychological, and Social Well-Being** as critical dimensions of flourishing; emphasizes that mental health and illness are separate but related continua. **​**  **Tripartite structure and specific dimensions reflecting positive mental health**   - **Emotional Well-Being:** - **Psychological Well-Being** - **Social Well-Being** | |  |
| 17.Addressing the Determinants of Positive Mental Health: Concepts, Evidence, and Practice (Barry,2009) (20).  International | Literature Review and Conceptual Analysis  **Positive Mental Health** | | **Aim** Explore determinants of positive mental health.  **Population**  **Global population** | Mental health is not just the absence of illness but includes **Emotional, Psychological, and Social Well-Being.**  Emphasizes the role of self-management, social support, and resilience in promoting Well-Being; highlights social determinants such as education, income, and social inclusion as critical factors for mental health  **Positive mental health usually encompasses:**   - **Emotional** - Physical Health - Spiritual Well-Being - **Eudaimonic Well-Being** | |  |
| 18. Positive Mental Well-Being: A Validation of a Rasch-Derived Version of the Warwick-Edinburgh Mental Well-Being Scale (WEMWBS)(Houghton et al.,2017) (21).  Australia | Validation of the Rasch-Derived Version of the Warwick-Edinburgh Mental Well-Being Scale  **Positive Mental Health** | | **Aim**: Validation of the Warwick-Edinburgh Mental Well-Being Scale (WEMWBS**)**  **Population**  General adult population (ages 18-69) in Western Australia. | The study confirmed that **positive mental well-being** involves both **Hedonic** and **Eudaimonic Well-Being.**  **Concept of Mental Well-Being**   - **Hedonic Well-Being** - **Eudaimonic Well-Being** | |  |
| 19.Psychological well-being revisited: Advances in the science and practice of eudemonia (Ryff,2014) (22).  United States | Literature Review and Conceptual Analysis  **Psychological Well-Being** | | **Aim**: To review advances in the understanding of Psychological Well-Being over the past 25 years, particularly focusing on eudaimonia (i.e., Well-Being through self-actualization, purpose, and personal growth) across the lifespan.  **Population**  Adults across various age groups, with a focus on understanding Well-Being throughout the adult lifespan. | Ryff identifies **six key dimensions of Psychological Well-Being:**   - Autonomy - Self-acceptance - Purpose of life - Environmental mastery - Personal growth - Positive relationship   **Additional Concepts**   - **Eudaimonic Well-Being** - **Hedonic Well-Being**: | |  |
| 20.Psychological wellbeing, health and aging (Steptoe et al.,2015) (23).  United Kingdom & United States | Quantitative Study using International Survey Data  **Psychological Well-Being/Subjective Well-Being** | | **Aim and Population**  To examine the relationship between Psychological Well-Being and physical health in older adults, using data from international surveys, such as the English Longitudinal Study of Ageing (ELSA) and the Gallup World Poll. | **Psychological Well-Being** and health are closely linked to age. **Three aspects of Psychological Well-Being**     - **Evaluative Well-Being** - **Hedonic Well-Being** - **Eudemonic Well-Being**   The relation between physical health and subjective Well-Being is bidirectional. | |  |

## **Legend:**

## This table provides an overview of the included studies in the concept analysis of Perinatal Mental Well-Being (PMWB). It summarizes key details and key aspects, as well as the **research design** and the **type of well-being assessed**. Additionally, the table outlines the **aim of each study**, details on the **study population**, and presents **key findings related to PMWB**, with a particular focus on how PMWB is **defined and conceptualized**. The theoretical insights derived from these studies contribute to a broader understanding of PMWB as a multidimensional construct.

## **Abbreviation**

PMWB: Perinatal Mental Well-Being, PWB: Perinatal Well-Being, GWB: General Well-Being; MWB: Mental Well-Being, WB: Well-Being

WEMWBS: Warwick-Edinburgh Mental Well-Being Scale

WiP: Well-Being in Pregnancy

SOC (Sense of Coherence)

## **References**

1. McGinley PD, ClinHypn, & Biotech, B. Capture my mood: A feasibility study to develop a visual scale for women to self-monitor their mental wellbeing following birth Evidence Based Midwifery. 2017;15(2):54-9.

2. Alderdice F, McNeill J, Gargan P, Perra O. Preliminary evaluation of the Well-being in Pregnancy (WiP) questionnaire2017. 1-10 p.

3. Mirzakhani K, Ebadi A, Faridhosseini F, Khadivzadeh T. Well-being in high-risk pregnancy: an integrative review. BMC Pregnancy and Childbirth. 2020;20:1-14.

4. Wadephul F, Glover L, Jomeen J. Conceptualising women's perinatal well-being: A systematic review of theoretical discussions. Midwifery. 2020;81:102598.

5. Allan C, Carrick-Sen D, Martin CR. What is perinatal well-being? A concept analysis and review of the literature. Journal of Reproductive and Infant Psychology. 2013;31(4):381-98.

6. Alderdice F, Gargan P. Exploring subjective wellbeing after birth: A qualitative deductive descriptive study2019.

7. Fen CM, Isa I, Chu CW, Ling C, Ling SY. Development and validation of a mental wellbeing scale in Singapore. Psychology. 2013;4(07):592.

8. Robitschek C, & Keyes, C. L. M. . Keyes's model of mental health with personal growth initiative as a parsimonious predictor. Journal of Counseling Psychology, 56(2), 321-329. 2009.

9. Huber M, van Vliet M, Giezenberg M, Winkens B, Heerkens Y, Dagnelie PC, et al. Towards a 'patient-centred' operationalisation of the new dynamic concept of health: a mixed methods study. BMJ open. 2016;6(1):e010091.

10. Huppert FA. Challenges in defining and measuring well-being and their implications for policy. Future directions in well-being: Education, organizations and policy. 2017:163-7.

11. Linton M-J, Dieppe P, Medina-Lara A. Review of 99 self-report measures for assessing well-being in adults: exploring dimensions of well-being and developments over time. BMJ open. 2016;6(7):e010641.

12. Keyes CL. Mental well-being: International contributions to the study of positive mental health: Springer; 2013.

13. Joshanloo M, Weijers D. A two-dimensional conceptual framework for understanding mental well-being. PloS one. 2019;14(3):e0214045.

14. Barry MM. Promoting Positive Mental Health and Well-Being: Practice and Policy. In: Keyes CLM, editor. Mental Well-Being: International Contributions to the Study of Positive Mental Health. Dordrecht: Springer Netherlands; 2013. p. 355-84.

15. Organization WH. Promoting Mental Health: Concepts, Emerging Evidence, Practice. Summary Report. A report of the World Health Organization, Department of Mental Health and Substance Abuse in collaboration with the Victorian Health Promotion Foundation and the University of Melbourne. Geneva, Switzerland: World Health Organization. . (2004a).

16. WHO. Constitution of the World Health Organization. 1946. Bulletin of the World Health Organization. 1946;80(12):983-4.

17. Organization WH. Measurement of and target-setting for well-being: an initiative by the WHO Regional Office for Europe: second meeting of the expert group: Paris, France, 25–26 June 2012. 2012.

18. WHO. Mental health. Maternal mental health. Geneva (CH): World Health Organization 2018 [Available from: http://www.who.int/mental_health/maternal-child/maternal_mental_health/en/.

19. Keyes C, editor Mental Health as a Complete State: How the Salutogenic Perspective Completes the Picture2014.

20. Barry MM. Addressing the determinants of positive mental health: concepts, evidence and practice. International Journal of Mental Health Promotion. 2009;11(3):4-17.

21. Houghton S, Wood L, Marais I, Rosenberg M, Ferguson R, Pettigrew S. Positive mental well-being: A validation of a Rasch-derived version of the Warwick-Edinburgh Mental Well-Being Scale. Assessment. 2017;24(3):371-86.

22. Ryff CD. Psychological well-being revisited: Advances in the science and practice of eudaimonia. Psychotherapy and psychosomatics. 2014;83(1):10-28.

23. Steptoe A, Deaton A, Stone AA. Subjective wellbeing, health, and ageing. Lancet (London, England). 2015;385(9968):640-8.
