# Supplementary material for: Exploring perinatal mental well-being: a concept analysis from conception to one year postpartum
Source: BMC Pregnancy Childbirth. 2025 Oct 10;25:1068. doi: 10.1186/s12884-025-08015-y (PMC12512377; doi:10.1186/s12884-025-08015-y)
Supplement: Supplementary file 2 — Supplementary Material 2. [file 12884_2025_8015_MOESM2_ESM.docx]

| **Additional file 2: Overview of Critical Appraisal Using JBI Tools and Alternative Evaluation for Non-Empirical Sources** | | | | | | | | |
| --- | --- | --- | --- | --- | --- | --- | --- | --- |
| **No.** | **Author(s) and Year** | **Study Type** | **Proposed JBI Critical Appraisal Checklist** | **Appraisal Conclusion Based on JBI Criteria** | **Reason JBI Checklist Not Applicable** | **Alternative Evaluation Approach (if applicable)** | **Conclusion Based on Alternative Evaluation** | **Methodological Comments** |
| 1 | McGinley et al., 2017 | Feasibility Study / Mixed-Methods | JBI Critical Appraisal Checklist for Analytical Cross-Sectional Studies | Moderate Quality |  |  |  | Small sample size, but well-designed feasibility study using validated visual tools and outcome measures. |
| 2 | Alderdice et al., 2017 | Feasibility Study / Mixed-Methods | JBI Critical Appraisal Checklist for Analytical Cross-Sectional Studies | Moderate Quality |  |  |  | Detailed psychometric analysis using factor analysis and internal consistency measures. Sample well-described and appropriate for instrument testing. However, confounding factors were not identified or addressed, and no strategies for managing them were reported. |
| 3 | Mirzakhani et al., 2020 | Integrative Review | JBI Critical Appraisal Checklist for Systematic Reviews and Research Syntheses | Moderate to High Quality |  |  |  | Review question, search strategy, and synthesis approach are appropriate and well-executed using the Whittemore and Knafl method. Methodological rigor is ensured through dual screening and JBI-based quality appraisal. However, no reporting on independent critical appraisal or publication bias. |
| 4 | Wadephul et al., 2020 | Systematic Review | JBI Critical Appraisal Checklist for Systematic Reviews and Research Syntheses | High Quality |  |  |  | Systematic review with clearly defined question, transparent inclusion and appraisal process, and appropriate thematic synthesis. All JBI criteria were met, except for formal assessment of publication bias, which was not addressed. Tentative model developed with strong implications for research and practice. |
| 5 | Allan et al., 2013 | Concept Analysis / Literature Review | Not applicable |  | Concept analysis using Walker and Avant framework; not empirical or systematic review based. | Conceptual clarity, theoretical consistency, and relevance to PMWB assessed. | Provides strong conceptual clarity and internal consistency; relevance to the construct of Perinatal Well Being (PMWB) is evident. | Acceptable theoretical contribution with clear attribute definition and structured application of Walker and Avant’s method. However, lacks empirical triangulation or validation. |
| 6 | Alderdice & Gargan, 2019 | Qualitative Study | JBI Critical Appraisal Checklist for Qualitative Research | Moderate to High Quality |  |  |  | Well-designed qualitative study with clear research aim, appropriate methodology, and strong participant representation. However, lacks explicit researcher reflexivity and philosophical positioning. |
| 7 | Chan et al., 2019 | Scale Development and Validation (Cross-Sectional) | JBI Critical Appraisal Checklist for Analytical Cross-Sectional Studies | High Quality |  |  |  | Robust sample size and multi-phase validation. Exposure and outcome measures were clearly defined. Strong internal consistency, construct validity and appropriate statistical analysis. Potential confounders were not formally modeled but addressed through representative sampling and demographic reporting. |
| 8 | Robitschek & Keyes, 2009 | Analytical Cross-Sectional Study | JBI Critical Appraisal Checklist for Analytical Cross-Sectional Studies | Moderate to High Quality |  |  |  | Strong statistical design using confirmatory factor analysis (CFA) and structural equation modeling (SEM). Validated instruments were used throughout, though internal consistency of some subscales was low, and confounding factors were not explicitly identified or controlled. |
| 9 | Huber et al., 2016 | Mixed-Methods (Qualitative + Cross-Sectional Survey) | JBI Checklist for Analytical Cross-Sectional Studies | High Quality |  | Optional: Partial application of JBI Qualitative Research Checklist for exploratory phase | Valid contribution: qualitative phase was exploratory and foundational; strong conceptual grounding. | Strong quantitative design using valid and well-constructed survey instrument. Multivariate analysis addressed key confounding variables. Qualitative phase added conceptual depth and indicator development, though lacked researcher reflexivity. |
| 10 | Huppert, 2013 | Theoretical / Conceptual Chapter | Not applicable |  | Non-empirical conceptual analysis; lacks data or structured methodology | Evaluation based on conceptual clarity, relevance, and theoretical coherence. | Strong conceptual contribution; offers a well-reasoned policy-oriented framework. | Non-empirical; theoretical insights based on expert synthesis and interpretation of existing evidence. |
| 11 | Linton et al., 2016 | Systematic Review (Narrative Synthesis) | JBI Critical Appraisal Checklist for Systematic Reviews and Research Syntheses | High Quality  Comprehensive and well-structured review with a clearly defined review question, systematic search strategy, appropriate inclusion criteria, and rigorous thematic synthesis. Although no formal assessment of publication bias was reported, the overall methodological quality remains strong.  . |  |  |  | Clear objectives, structured systematic search and thematic synthesis. Dual data extraction and interdisciplinary review process enhanced credibility. Slight limitation: publication bias was not explicitly assessed. |
| 12 | Keyes, 2013 | Theoretical Review | Not applicable | Not applicable | No empirical data or systematic methodology | Evaluation based on conceptual clarity, relevance, and theoretical consistency | Strong conceptual contribution with a clear theoretical framework for positive mental health | Theoretical synthesis; no empirical data or formal quality appraisal. The work offers valuable conceptual insight into well-being. |
| 13 | Joshanloo & Weijers, 2019 | Scale Structure Analysis using MDS / Analytical Cross-Sectional Study | JBI Critical Appraisal Checklist for Analytical Cross-Sectional Studies | High Quality |  |  |  | Large multi-country study using validated well-being instruments and Multidimensional Scaling (MDS) to empirically derive a two-dimensional model. Robust statistical fit and theoretical interpretation across diverse cultural samples. No explicit adjustment for confounders. |
| 14 | Barry, 2017 | Policy Analysis | Not applicable | Not applicable | No original study; policy document | Evaluated based on clarity, scope, and relevance to PMWB | Well-structured policy analysis providing practical guidance and international context for mental health promotion. | Non-empirical conceptual analysis offering relevant policy insights. No systematic methodology or formal data appraisal applied. |
| 15 | WHO (1946, 2004, 2012, 2018) | Policy Reports | Not applicable | Not applicable | Institutional reports; no empirical framework | Assessed for authority, credibility, and conceptual contribution | Strong conceptual and policy-relevant contribution. WHO reports outline a globally recognized multidimensional model of health integrating physical, mental, and social well-being, promoting a shift from disease treatment to health promotion. | Non-empirical WHO reports grounded in policy development and public health vision. Rich in theoretical insights and holistic health frameworks, but no formal research methodology applied. |
| 16 | Keyes, 2014 | Integrated Theoretical and Empirical Analysis | JBI Critical Appraisal Checklist for Analytical Cross-Sectional Studies (adapted) | High Quality |  |  |  | Theory-driven review supported by robust empirical findings from large-scale longitudinal and twin studies. Includes statistical analyses, heritability models, and public health data. Offers a strong conceptual and empirical case for the dual continua model and mental health promotion strategies. |
| 17 | Barry, 2009 | Narrative Review / Theoretical Overview | Not applicable | Not applicable | No formal empirical study or data collection; theoretical and narrative synthesis | Evaluated based on conceptual clarity, integration of evidence, and relevance to mental health promotion | Rich conceptual contribution synthesizing multiple frameworks and empirical findings. Strong practical and policy relevance. | Narrative and conceptual synthesis, not a systematic review, but provides valuable multi-level insights into determinants of positive mental health |
| 18 | Houghton et al., 2017 | Scale validation | JBI Critical Appraisal Checklist for Analytical Cross-Sectional Studies | High Quality – Clear inclusion criteria, detailed setting, appropriate Rasch validation and statistical methods. |  |  |  | High-quality validation study using advanced psychometric techniques (Rasch modeling). The study demonstrates clear design, valid and reliable measures, appropriate handling of differential item functioning (DIF), and rigorous statistical analysis |
| 19 | Ryff, 2014 | Narrative Review / Theoretical Reflection | Not applicable | Not applicable | No empirical data or systematic methodology | Evaluation based on conceptual clarity, theoretical coherence, and relevance to PMWB | Comprehensive conceptual overview of eudaimonia and psychological well-being; strong theoretical synthesis relevant to health promotion practice. | Theoretical contribution synthesizing decades of research. Lacks formal empirical methodology, but provides influential conceptual insights and directions for future research and application |
| 20 | Steptoe et al., 2015 | Analytical Cross-Sectional (with longitudinal elements) | JBI Critical Appraisal Checklist for Analytical Cross-Sectional Studies | High Quality – Large population datasets, validated indicators, confounders identified and adjusted, and robust statistical analyses. |  |  |  | Strong epidemiological design using ELSA and Gallup datasets. Comprehensive statistical modelling with appropriate control for confounding factors. Clear operationalization of psychological well-being indicators. Includes both evaluative, hedonic and eudaimonic well-being, enhancing conceptual completeness. |
